# Differential inhibition of intra- and inter-molecular protease cleavages by antiviral compounds

**DOI:** 10.1128/jvi.00928-23

**Published:** 2023-12-04

**Authors:** Jennifer S. Doherty, Karla Kirkegaard

**Affiliations:** 1 Department of Genetics, Stanford University, Palo Alto, California, USA; 2 Department of Microbiology and Immunology, Stanford University, Palo Alto, California, USA; St. Jude Children's Research Hospital, Memphis, Tennessee, USA

**Keywords:** polyprotein, protease, antiviral, poliovirus, enterovirus A71, enterovirus D68, picornavirus, positive-stranded RNA virus, viral genetics, drug resistance

## Abstract

Enteroviruses encode two protease active sites, in the 2A and 3C coding regions. While they target many host proteins, they first need to be excised from the viral polyprotein in which they are embedded. Polyprotein cleavage can occur either intra-molecularly (in *cis*) or inter-molecularly (in *trans*). Previous work suggested that antivirals targeting intra-molecular cleavages could generate inhibitory precursors that can suppress the outgrowth of drug-resistant variants. Therefore, we wanted to evaluate enteroviral cleavage patterns to identify such obligate intra-molecular cleavages as a method of drug target selection. Using translation extracts, we show that *cis* cleavage of the 2A protease N-terminal junction is conserved across three enterovirus clades, while the mechanism for the N-terminal junction of 3C varies, with enterovirus D68 3C cleavage occurring in *cis* and poliovirus 3C cleavage occurring in *trans*. Antiviral agents targeting proteases are generally identified via their ability to block the cleavage of artificial peptide substrates. Here, we show that antivirals identified for their ability to block inter-molecular cleavage can sometimes block intra-molecular cleavage of the protease from its polyprotein but with widely varying efficacy. Additionally, we demonstrate that for three enteroviral species, genomes defective in 2A protease activity suppress the growth of wild-type virus in mixed populations, supporting the hypothesis that preventing intra-molecular cleavage at the VP1·2A junction can create dominantly inhibitory precursors. These data argue that, to reduce the likelihood of drug resistance, protease-targeted antivirals should be evaluated for their ability to block intra-molecular polyprotein cleavages in addition to inter-molecular cleavage of other substrates.

**IMPORTANCE:**

Most protease-targeted antiviral development evaluates the ability of small molecules to inhibit the cleavage of artificial substrates. However, before they can cleave any other substrates, viral proteases need to cleave themselves out of the viral polyprotein in which they have been translated. This can occur either intra- or inter-molecularly. Whether this process occurs intra- or inter-molecularly has implications for the potential for precursors to accumulate and for the effectiveness of antiviral drugs. We argue that evaluating candidate antivirals for their ability to block these cleavages is vital to drug development because the buildup of uncleaved precursors can be inhibitory to the virus and potentially suppress the selection of drug-resistant variants.

## INTRODUCTION

Enteroviruses (EVs) constitute a genus of positive-stranded RNA viruses that includes significant human pathogens such as poliovirus, rhinovirus, EV-A71, and EV-D68. They form part of the larger *Picornaviridae* family. Although poliovirus has been largely eradicated, vaccine-derived cases still emerge (1–5) and have recently caused outbreaks in Israel, the UK, and the USA (6). EV-A71 can cause severe neurological outcomes in children (7–9), and EV-D68 has been associated with acute flaccid myelitis among children in recent years (10–13). There are currently no vaccines or antivirals available for the treatment of these emerging EV species.

**Fig 1:**
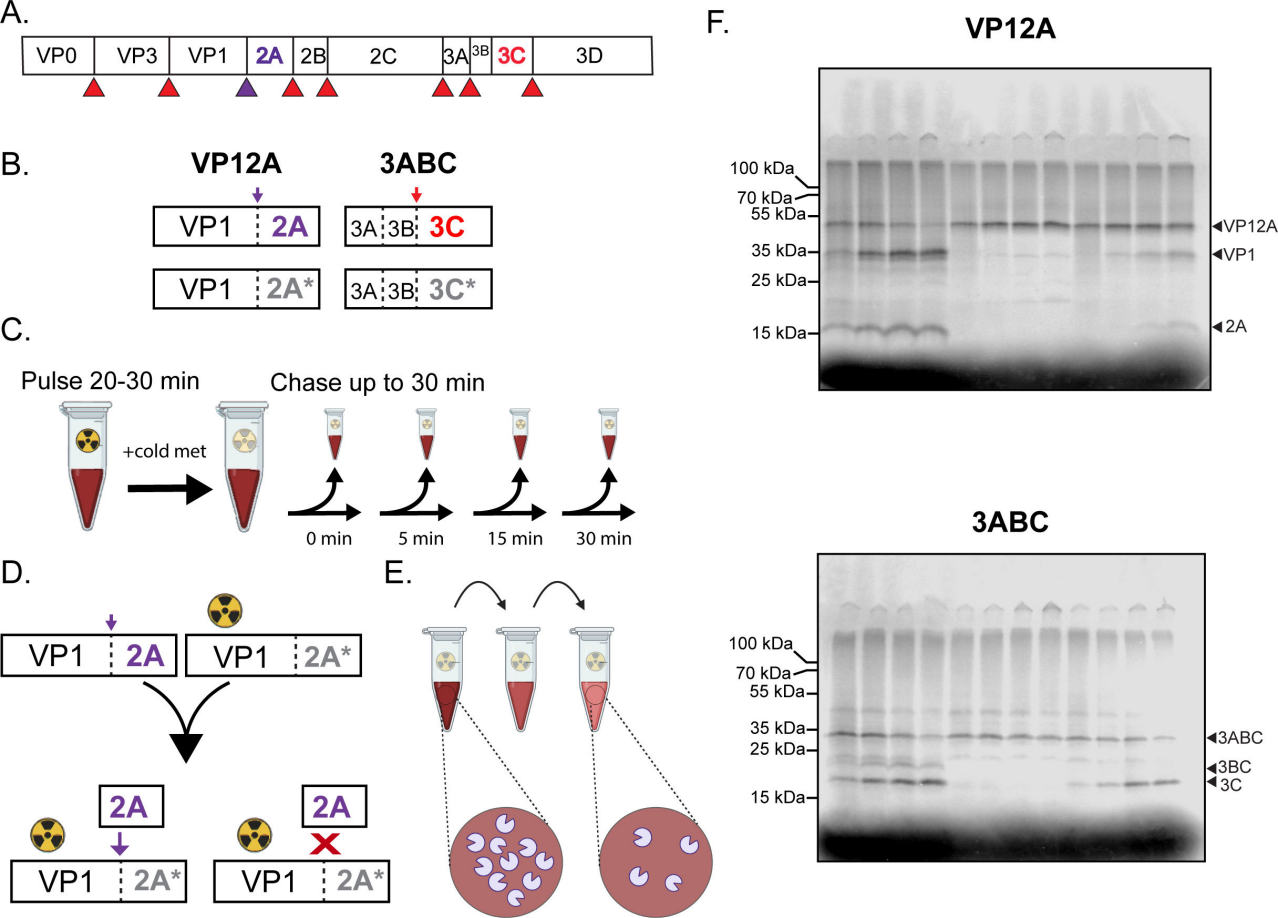
Design of polyprotein processing assays. (**A**) EV polyprotein showing cleavages made by 3C protease activity (red) and 2A protease activity (purple). (**B**) Constructs used to program translation extracts from rabbit reticulocytes. 2A* and 3C* represent catalytically inactive mutant proteins 2A C109R and 3C C147R. (**C**) Pulse-chase experimental design. (D) *Trans* cleavage assay design. Catalytically active, unlabeled protease is combined with catalytically inactive protein that has been radioactively labeled. If *trans* cleavage can occur, the inactive precursor will be processed (left), and if it cannot, no processing will occur (right). (E) Dilution sensitivity assay design. If processing occurs in *cis*, the rate of the reaction should not change upon dilution. (**F**) Representative uncropped gels of poliovirus VP1·2A (top) and 3ABC (bottom) *trans* cleavage assay with molecular weight markers indicated.

All members of the EV family share the same genomic architecture. These genomes are translated as polyproteins that must be processed into individual viral gene products by two virally encoded proteolytic activities. 3C and 3CD make most of the cleavages while 2A cleaves at the border between capsid protein VP1 and its own coding region (Fig. 1A). Both proteases also cleave many cellular targets to make the cell more hospitable for viral replication (14–17), including eukaryotic initiation factor 4G (eIF4G) by 2A, whose cleavage stimulates the preferential translation of viral proteins during infection (18). Although enteroviral proteases have been extensively studied as drug targets, no enteroviral protease inhibitors have been approved for clinical use. A deeper understanding of EV biology could serve as a basis for more rational drug design for all viruses that utilize a polyprotein strategy.

Release of polyprotein-embedded proteases can occur either intra-molecularly, with a single molecule cleaving itself, or inter-molecularly, with one molecule cleaving a second molecule. These mechanisms can also be referred to as *cis* (intra-molecular) and *trans* (inter-molecular). In positive-stranded RNA virus groups such as flaviviruses (19, 20) and alphaviruses (21, 22), some of the polyprotein cleavages have been shown to occur exclusively in *cis*, enabling rapid excision from the polyprotein and providing a unique opportunity for antiviral development. RNA virus populations exist as a quasispecies (23). Therefore, drug-susceptible and newly minted drug-resistant variants coexist in the same cell and can interact. In dengue virus, mutant genomes encoding proteins that fail to make an obligate *cis* cleavage were shown to have an inhibitory effect on wild-type (WT) viral growth in mixed transfections (20), suggesting that blocking such cleavages could lead to the production of precursors that act as dominant inhibitors. Therefore, identification of intra-molecular cleavages in viral proteins will reveal promising substrates for antiviral targeting.

In the *EV* genus, poliovirus polyprotein processing has been the most intensively studied. While some work on poliovirus 2A has suggested that its excision from the polyprotein occurs exclusively intra-molecularly (24, 25), other data suggested that this proteolytic event could occur in *trans* (26, 27). For 3C and 3CD proteases, inter-molecular processing of polyprotein precursors has been extensively investigated (28–31), but the mechanism of processing for the 3C N-terminal junction itself is unresolved. Some work in poliovirus suggested that this processing could occur inter-molecularly (30, 32, 33), while work in encephalomyocarditis virus (EMCV), a non-EV member of the *Picornaviridae*, showed that 3C self-processing was dilution insensitive, and therefore likely occurs intra-molecularly (34–36).

Protease inhibitors are often identified by screening for the ability to block the cleavage of small peptide substrates in *trans*. However, intra-molecular cleavages display different kinetics and may, therefore, serve as unique drug targets. In this work, we have identified several intra-molecular cleavages in the poliovirus and EV-D68 protease processing cascades and evaluated the efficacy of existing small molecules on both intra- and inter-molecular protease cleavages. We also show, for three different EVs, that specifically blocking VP1·2A cleavage in a mixed infection can suppress the growth of all viruses in the same cell, even those that encode functional 2A protease. Thus, drugs inhibiting these intra-molecular cleavages may be especially effective due to their ability to suppress the emergence of resistant variants.

## RESULTS

To identify intra- and inter-molecular cleavages in EV polyprotein processing, we developed constructs encoding short regions of the viral polyprotein that included the protease of interest. These constructs were used to program translation extracts (Fig. 1B), and the translation products were then used as substrates in pulse-chase reactions (Fig. 1C). To assay *trans* cleavage directly, constructs encoding catalytically inactive protease were translated in the presence of radioactive label. In the absence of enzymatic activity, the product is predicted to remain a full-length precursor (Fig. 1D, right). If *trans* processing can occur, the radioactively labeled precursor will be processed upon the addition of an active, unlabeled protease (Fig. 1D, left). Thus, if no processing is observed, we can conclude that the precursor is cleaved exclusively intra-molecularly under these conditions. It is not possible to measure *cis* cleavage alone because multiple copies of a protein are translated, so *trans* cleavage is always a possibility. Therefore, to confirm the mechanism of *cis* cleavages, we can test the dilution sensitivity of the processing of WT construct. To this end, catalytically active construct translated with radioactive label is diluted during the chase period (Fig. 1E). Upon dilution, the rate of intra-molecular reactions should be unchanged while inter-molecular reactions are predicted to occur more slowly.

Example translations for poliovirus VP1·2A and 3ABC are shown in Fig. 1F. For VP1·2A processing, we observed bands at the expected sizes of VP1·2A precursor plus cleavage products VP1 and 2A (Fig. 1F, top; Fig. S1, right; Table S1). For 3ABC processing, we observed bands at the expected sizes of the 3ABC precursor, 3BC, and 3C (Fig. 1F, bottom; Fig. S1, left; Table S1). Bands corresponding to 3A or 3AB were not observed because they ran with the unincorporated radioactive methionine at the bottom of the gels, while the accumulation of 3BC may reflect an alternative processing pathway for P3 that has also observed during infection (30, 37).

### Poliovirus 2A protease cleaves its N-terminal junction primarily intra-molecularly

**Fig 2:**
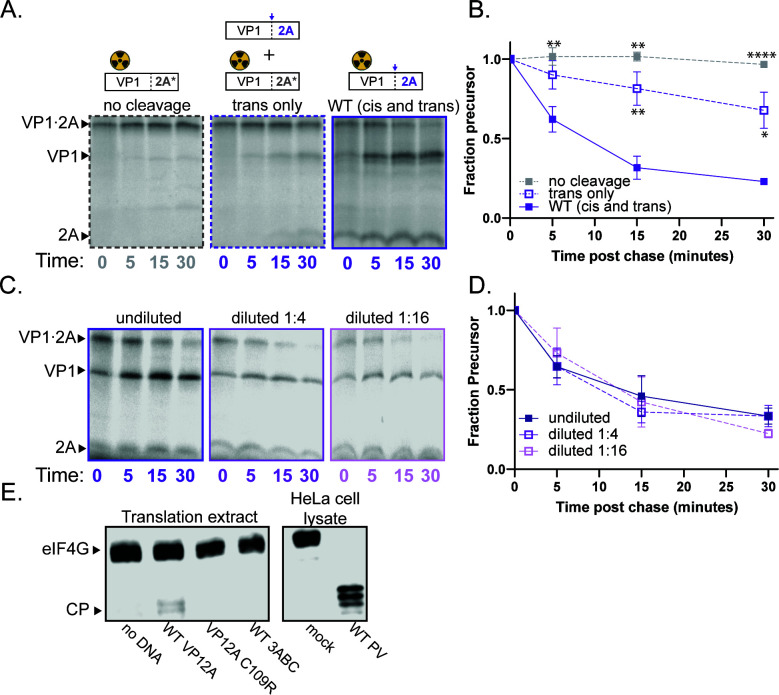
Poliovirus 2A processing occurs in *cis*. (**A**) Representative gels for poliovirus 2A *trans* cleavage assay. 2A* represents the C109R active site mutation. No cleavage (left): C109R construct with unlabeled WT 3ABC (negative control). *Trans*-only (middle): C109R construct with unlabeled WT VP1·2A. WT (*cis* and *trans*) (right): catalytically active VP1·2A. (**B**) Quantification of *trans* cleavage assay. Fraction precursor is calculated by dividing the intensity of the VP1·2A band by the sum of the VP1·2A and the VP1 band intensities and is normalized to the value at time 0. (**C**) Representative gels for poliovirus 2A dilution sensitivity assay. (D) Quantification of VP1·2A dilution sensitivity assay. Data were quantified as in B. (**E**) Western blot of eIF4G from translation extracts programmed with the indicated RNA (left) or cell lysates infected with poliovirus (right).

To determine whether the excision of the N-terminus of 2A protease occurs in *cis* or in *trans*, we used a construct encoding only VP1·2A to program translation extracts. Representative images for the *trans* cleavage assay are shown in Fig. 2A. As expected, VP1·2A with a mutation in the 2A active site was not processed (left panel). In the presence of catalytically active, unlabeled VP1·2A, processing was incomplete (center panel). In contrast, self-processing of the WT construct, which can occur either in *cis* or in *trans*, was rapid and complete (right panel). These data are quantified in Fig. 2B and argue that the primary mode of cleavage for the VP1·2A junction is intra-molecular under these conditions. This is confirmed by the results of the dilution sensitivity assay that shows that the rate of precursor processing does not change upon dilution (Fig. 2C and D). To verify that the 2A protease can cleave in *trans* under the assay conditions, we immunoblotted for eIF4G, which is naturally present in the translation extracts. In poliovirus-infected cell lysates, eIF4G cleavage was complete by 5 hours after infection (Fig. 2E, Lane 5). In the programmed translation reactions, only the catalytically active 2A construct could cleave eIF4G (Fig. 2E, Lane 2).

### Poliovirus 3C protease cleaves its N-terminal junction primarily inter-molecularly

**Fig 3:**
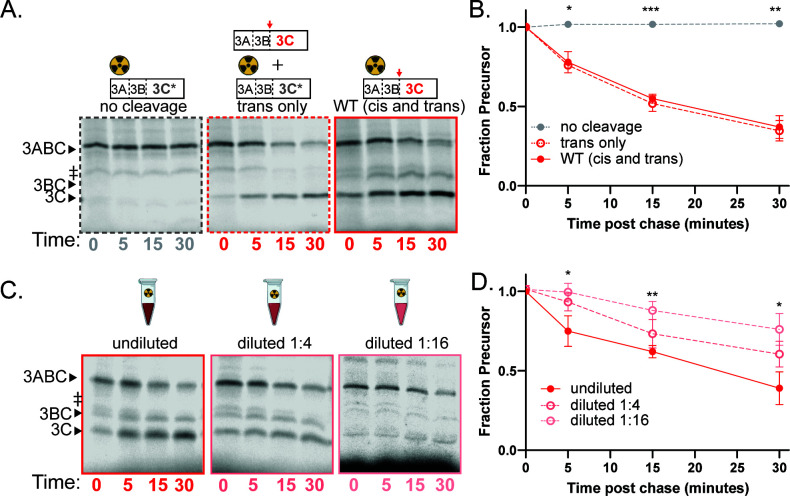
Poliovirus 3C processing occurs primarily in *trans*. (**A**) Representative gels for poliovirus 3C *trans* cleavage assay. 3C* represents the C147R active site mutation. No cleavage (left): poliovirus 3C C147R with unlabeled WT VP1·2A (negative control); *trans* only (middle): 3C C147R with unlabeled catalytically active 3ABC. WT (*cis* and *trans*) (right): catalytically active 3ABC. ǂ represents an aberrant translation product due to internal translation initiation (38). (B) Quantification of *trans* cleavage assay. Fraction precursor is calculated by dividing the intensity of the 3ABC band by the sum of the 3ABC and the 3C band intensities and is normalized to the value at time 0. (**C**) Representative gels for poliovirus 3C dilution sensitivity assay. (D) Quantification of dilution sensitivity assay. Data were quantified as in B.

To determine whether the excision of the N-terminus of 3C protease occurs in *cis* or in *trans*, we also monitored its processing by pulse chase in translation exacts. Representative gels for the *trans* cleavage assay are shown in Fig. 3A and quantified in Fig. 3B. Processing of 3ABC occurred primarily between 3B and 3C. The rate of processing was identical in the *trans*-only reaction and in the WT reaction where both *cis* and *trans* cleavages could occur (Fig. 3A and B). Additionally, the rate decreased with increasing dilution (Fig. 3C and D). Both findings are characteristics of an inter-molecular cleavage pattern. In all assays with labeled WT precursor (Fig. 3A, right; Fig. 3C), a band corresponding to 3BC is also produced. This band is not seen in the *trans* cleavage condition (Fig. 3A, middle). Thus, cleavage from 3ABC to 3BC is made in these extracts intra-molecularly but only inefficiently. An additional slightly larger band was also observed on many of our poliovirus 3ABC gels (Fig. 3A and C, ǂ) and is likely the result of inappropriate translation initiation that sometimes occurs in rabbit reticulocyte lysates (38).

### The 2A cleavage mechanism is conserved in multiple EVs, while the 3C mechanism differs

**Fig 4:**
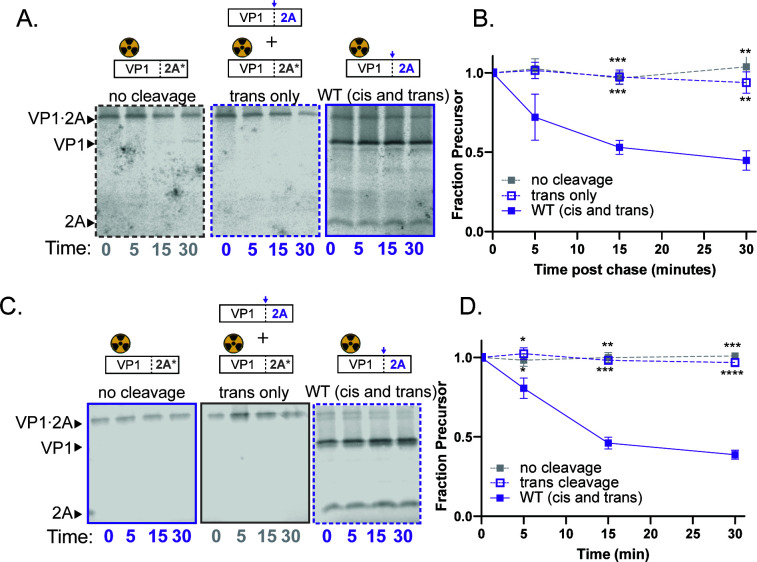
EV-D68 and EV-A71 self-processing by 2A occur in *cis*. Representative gels (**A**) and quantification (**B**) for EV-D68 VP1·2A *trans* cleavage assay. 2A* represents the C107R active site mutation. No cleavage: EV-D68 2A C107R with unlabeled 3ABC (negative control). *Trans* only: EV-D68 2A C107R with unlabeled catalytically active VP1·2A; WT (*cis* and *trans*): EV-D68 catalytically active VP1·2A. (**C and D**) Representative gels and quantification for EV-A71 VP1·2A *trans* cleavage assay. 2A* represents C110A active site mutation. No cleavage: EV-A71 2A C110A with unlabeled 3ABC (negative control). *Trans* only: EV-A71 2A C110A with unlabeled catalytically active VP1·2A. WT (*cis* and *trans*): EV-A71 catalytically active VP1·2A. Data were quantified as in Fig. 2.

To determine how conserved these mechanisms are across the *EV* genus, we established the *cis* and *trans* cleavage efficiencies for EV-D68 and EV-A71 2A proteases and for EV-D68 3C protease. In the *trans* cleavage assay for 2A protease, no inter-molecular processing of EV-D68 VP1·2A was observed, although processing occurred rapidly for the active precursor (Fig. 4A and B). Exclusively intra-molecular cleavage was also observed for the VP1·2A precursor of EV A71 (Fig. 4C and D), arguing that rapid intra-molecular cleavage between the capsid proteins and 2A protease is likely to be conserved across the *EV* genus.

**Fig 5:**
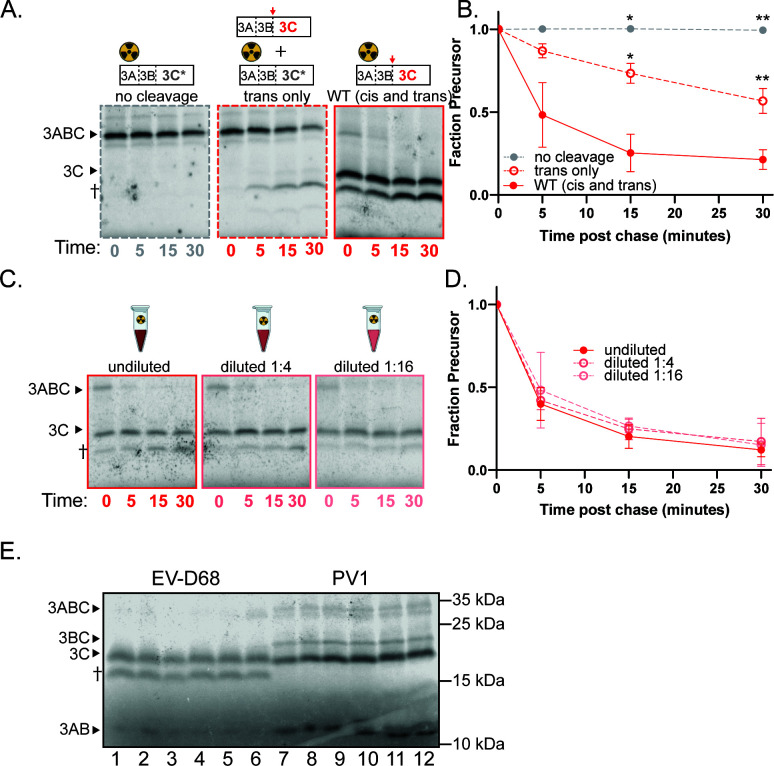
EV-D68 3C protease self-processing occurs in *cis*. Representative gels (**A**) and quantification (**B**) of EV-D68 3ABC *trans* cleavage assay. 3C* represents the C147R active site mutation. No cleavage: EV-D68 3C C147R with unlabeled VP1·2A (negative control). *Trans* cleavage: EV-D68 3C C147R with unlabeled catalytically active 3ABC; WT (*cis* and *trans*): EV-D68 catalytically active 3ABC. † indicates unidentified cleavage product. Fraction precursor is calculated by dividing the intensity of the 3ABC band by the sum of the 3ABC, 3C, and † band intensities and is normalized to the value at time 0. (**C**) Representative gels of EV-D68 3ABC dilution sensitivity assay. (**D**) Quantification of EV-D68 3ABC dilution sensitivity assay. Quantified as in B. (**E**) EV-D68 3ABC (Lanes 1–6) and poliovirus 3ABC (Lanes 7–12) were translated with subinhibitory concentrations of rupintrivir to compare the effects between viruses. Gel is included to show that the band represented by † is too small to be 3C.

The processing of 3ABC precursors, however, differed between EV-D68 and poliovirus. Unlike the effective *trans* cleavage observed with poliovirus 3ABC (Fig. 3A and B), EV-D68 3ABC processing in the *trans*-only cleavage condition occurred much more slowly and incompletely (Fig. 5A and B) implying that the cleavage occurs primarily intra-molecularly. The dilution sensitivity assay, which showed that the rate of EV-D68 3ABC processing did not change upon dilution (Fig. 5C and D), corroborates this interpretation.

**Fig 6:**
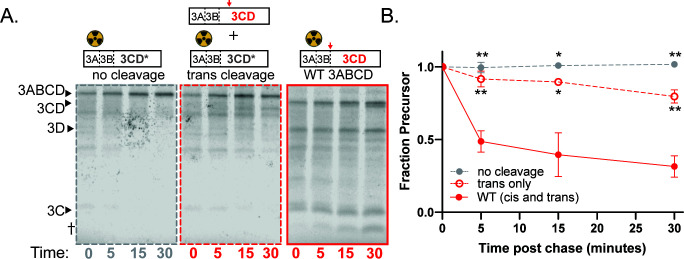
EV-D68 3ABCD self-processing occurs in *cis*. (**A**) Representative gels of 3ABCD *trans* cleavage assay. 3CD* represents the 3C C147R catalytically inactive mutant. No cleavage: EV-D68 3C C147R construct with unlabeled VP1·2A (negative control); *trans* only: EV-D68 3C C147R with unlabeled catalytically active 3ABCD. WT (*cis* and *trans*): EV-D68 catalytically active 3ABCD. (**B**) Quantification of EV-D68 3ABCD *trans* cleavage assay. Fraction precursor is calculated by dividing the intensity of the 3ABCD band by the sum of the 3ABCD, 3CD, and 3D band intensities and is normalized to the value at time 0.

EV-D68 3ABC processing also produced an additional band of approximately 16 kDa that does not correspond to the size of any identified 3ABC cleavage products and is of unknown origin (Fig. 5E). The production of this band requires 3C protease activity, as it is not present in translation extracts programmed with catalytically inactive 3C (Fig. 5A and C) or when 3C is inhibited by an antiviral drug (Fig. 8A). It is also produced when full-length 3ABCD is allowed to self-process (Fig. 6A). Interestingly, this unknown band is the major cleavage product when EV-D68 3ABC cleavage is forced to occur in *trans*, highlighting the importance of intra-molecular cleavage reactions for proper polyprotein processing.

Because 3C and 3CD exhibit different substrate recognition properties, we wanted to determine whether 3ABCD precursor processing exhibited different kinetics from 3ABC processing. Extracts programmed with EV-D68 3ABCD constructs showed a strong preference for *cis* processing, with WT cleavage so rapid that little full-length precursor was observed at the beginning of the chase period (Fig. 6). This argues that the rapid intra-molecular cleavage observed by the EV-D68 3ABC precursor is not an artifact of using a truncated portion of the P3 polyprotein but rather may be a biological feature of the activity of this protease.

### Antivirals have varying efficacy in inhibiting intra-molecular cleavages

Proteases of positive-stranded viruses have two types of substrates during infection: host proteins, which must be cleaved inter-molecularly, and viral polyproteins, which can be cleaved either intra-molecularly or inter-molecularly. Few studies have been done to correlate protease inhibitor efficacy against viruses with the type of cleavage that is being inhibited. To determine how effectively the different cleavage events that occur during poliovirus and EV-D68 infections are inhibited by existing protease inhibitors, we investigated the effects of 2A inhibitors telaprevir and zVAM.fmk and 3C inhibitor rupintrivir on the inter- and intra-molecular cleavages described here.

**Fig 7:**
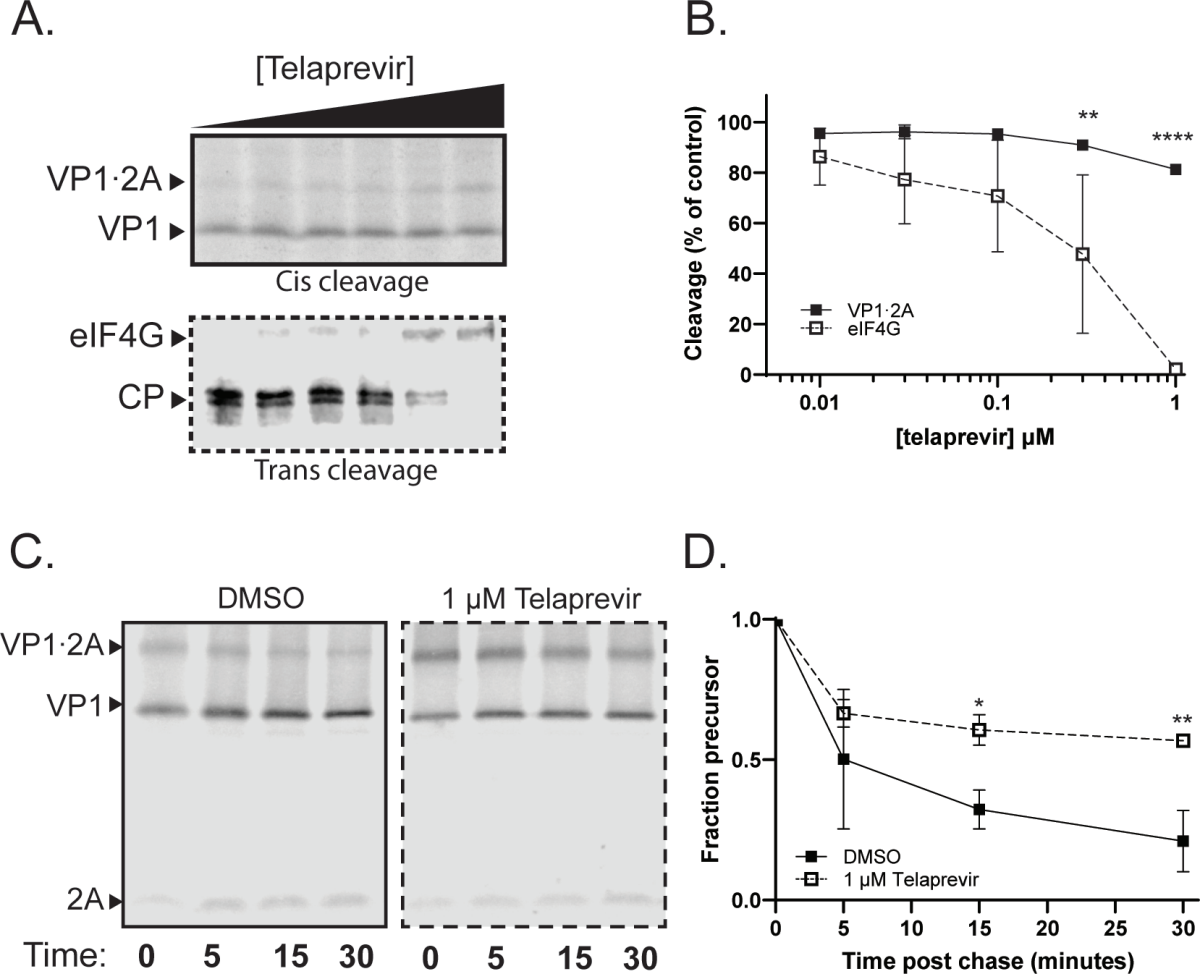
Telaprevir can inhibit the intra-molecular cleavage of EV-D68 2A. (**A**) Representative gels of self-processing (top panel) and eIF4G cleavage (bottom panel) by EV-D68 2A in the presence of increasing concentrations of telaprevir. Telaprevir was added at the initiation of translation. CP, eIF4G cleavage products. (B) Quantification of telaprevir concentration curves. Percent cleavage normalized to the amount of cleavage in the DMSO condition (first lane). (**C and D**) Representative gels (**C**) and quantification (**D**) of EV-D68 VP1·2A pulse-chase experiment in the presence of telaprevir. Data were quantified as in Fig. 2.

Telaprevir, originally identified as an inhibitor of hepatitis C virus NS3/NS4A (39), was recently shown to be a potent and specific inhibitor of EV-D68 2A protease, with reported EC_50_ values for EV-D68 viral growth ranging from 0.5 to 2.0 µM (40). To compare the ability of this small molecule to inhibit cleavage of inter-molecular targets, for which it was identified, with its ability to inhibit intra-molecular self-processing, an EV-D68 VP1·2A construct was translated in the presence of increasing concentrations of telaprevir (Fig. 7A, top). To determine the effect of telaprevir on inter-molecular cleavage of host eIF4G, a construct encoding 2A protease alone was translated without radioactive methionine, and the proportion of intact eIF4G within the translation extract was ascertained via immunoblot (Fig. 7A, bottom). At 1-µM telaprevir, the maximum concentration we could test in our translation extracts, the intra-molecular processing of VP1·2A was only 20% inhibited, while the inter-molecular processing of eIF4G was fully inhibited (Fig. 7B). Based on these data, the approximate IC_50_ for the intra-molecular cleavage of VP1·2A is around 4 µM. The drug is more potent in blocking the inter-molecular cleavage of eIF4G, with an IC_50_ of approximately 200 nM (Table 1). To characterize the inhibition of EV-D68 VP1·2A self-processing by telaprevir further, pulse-chase experiments were performed to monitor the kinetics of precursor processing in the presence and absence of 1 µM of the drug (Fig. 7C). In the presence of telaprevir, the rate of VP1·2A self-processing was significantly decreased for the WT construct (Fig. 7D) but not for a telaprevir resistant mutant (Fig. S2), confirming that the reduction in VP1·2A self-processing, although modest, is due to inhibition of 2A activity.

**TABLE 1:** Approximate IC_50_ values for intra- and inter-molecular cleavages by protease-targeted antivirals

Drug	Cleavage	IC_50_ (µM)	95% CI (µM)
Telaprevir	EV-D68 VP1·2A (intra-molecular)	3.96	3.17–5.12
	eIF4G (inter-molecular)	0.19	0.099–0.356
zVAM.fmk	Poliovirus VP1·2A (intra-molecular)	1570	1320–1890
	eIF4G (inter-molecular)	18.9	12.6–27.3
Rupintrivir	EV-D68 3ABC (intra-molecular)	0.14	0.146–0.193
	Poliovirus 3ABC (inter-molecular)	0.76	0.584–0.988

**Fig 8:**
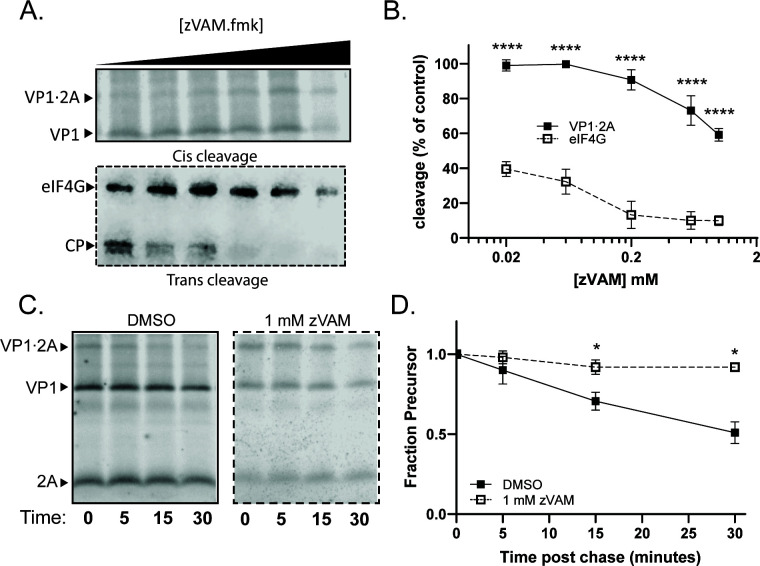
zVAM.fmk can inhibit self-processing of poliovirus 2A. (**A**) Representative gels of self-processing (top panel) and eIF4G cleavage (bottom panel) by poliovirus 2A in the presence of increasing concentrations of zVAM.fmk. zVAM.fmk was added at the initiation of translation. CP, eIF4G cleavage products. (**B**) Quantification of zVAM.fmk concentration curves. Percent cleavage normalized to the amount of cleavage in the DMSO condition (first lane). (**C and D**) Representative gels (**C**) and quantification (**D**) of poliovirus VP1·2A pulse chase in the presence of 1-mM zVAM.fmk.

Peptide inhibitor zVAM.fmk was initially identified as a rhinovirus protease inhibitor. It was shown to inhibit both rhinovirus VP1·2A precursor processing and eIF4G cleavage with optimal effects at 200 µM (41). To determine whether zVAM.fmk also inhibits poliovirus 2A protease, translation extracts programmed with poliovirus VP1·2A were incubated with increasing concentrations of zVAM.fmk (Fig. 8A). Again, intra-molecular cleavage of VP1·2A, while significantly inhibited by zVAM.fmk, was much less affected than the inter-molecular cleavage of eIF4G (Fig. 8A and B). The calculated IC_50_ for intra-molecular VP1·2A cleavage is around 1.6 mM, while inhibition of inter-molecular eIF4G cleavage was much more effective, with an IC_50_ of approximately 20 µM (Table 1). Pulse-chase experiments also revealed significant inhibition of VP1·2A *cis* cleavage at high concentrations (Fig. 8C and D) confirming the compound’s efficacy but low potency for this intra-molecular reaction.

**Fig 9:**
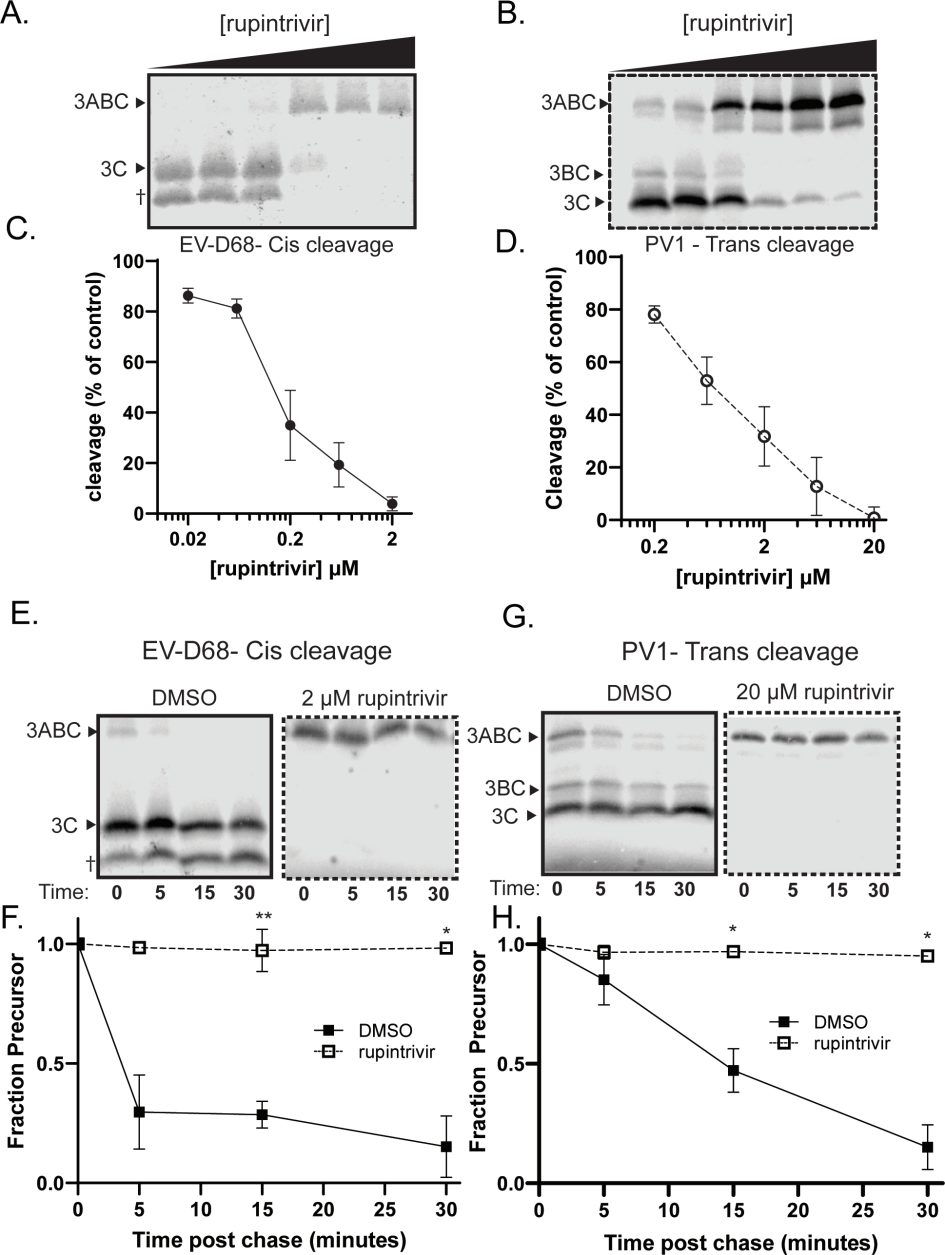
Rupintrivir inhibits 3ABC self-processing in poliovirus and EV-D68. (**A and B**) Representative gels of rupintrivir concentration curve in EV-D68 (**A**) and poliovirus (**B**). Rupintrivir was added at the initiation of translation. (**C and D**) Quantification of A and B. Percent cleavage normalized to the amount of cleavage in the DMSO condition (first lane). (**E and F**) Representative gel and quantification of EV-D68 pulse-chase experiment in the presence of 2-µM rupintrivir. (**G and H**) Representative gel and quantification of poliovirus pulse-chase experiment in the presence of 20-µM rupintrivir.

Rupintrivir was identified as an inhibitor of rhinovirus 3C protease (42) but has since been shown to inhibit the enzymatic activity of a wide variety of viral 3C and 3C like proteases (43–47). It is an efficacious inhibitor of viral growth as well: the reported EC_50_ for EV-D68 isolates ranges from 2 to 3 nM (44), while, for poliovirus, the reported EC_50_ ranges from 5 to 40 nM (48). In translation extracts, we observed near-complete inhibition of both EV-D68 and poliovirus 3ABC processing, albeit at different concentrations (Fig. 9A to D). The IC_50_ for the intra-molecular cleavage of EV-D68 3ABC is approximately 140 nM, while the IC_50_ for the inter-molecular cleavage of poliovirus 3ABC is approximately 760 nM (Table 1). In this case, the intra-molecular cleavage of EV-D68 3ABC is approximately fivefold more susceptible to rupintrivir than the inter-molecular cleavage of poliovirus 3ABC, which correlates with the relative effectiveness of rupintrivir against viral replication. Upon pulse chase, we observed essentially 100% inhibition of self-processing for both EV-D68 intra-molecular processing (Fig. 9E and F) and poliovirus inter-molecular processing (Fig. 9G and H).

### Inhibition of 2A protease is genetically dominant

**Fig 10:**
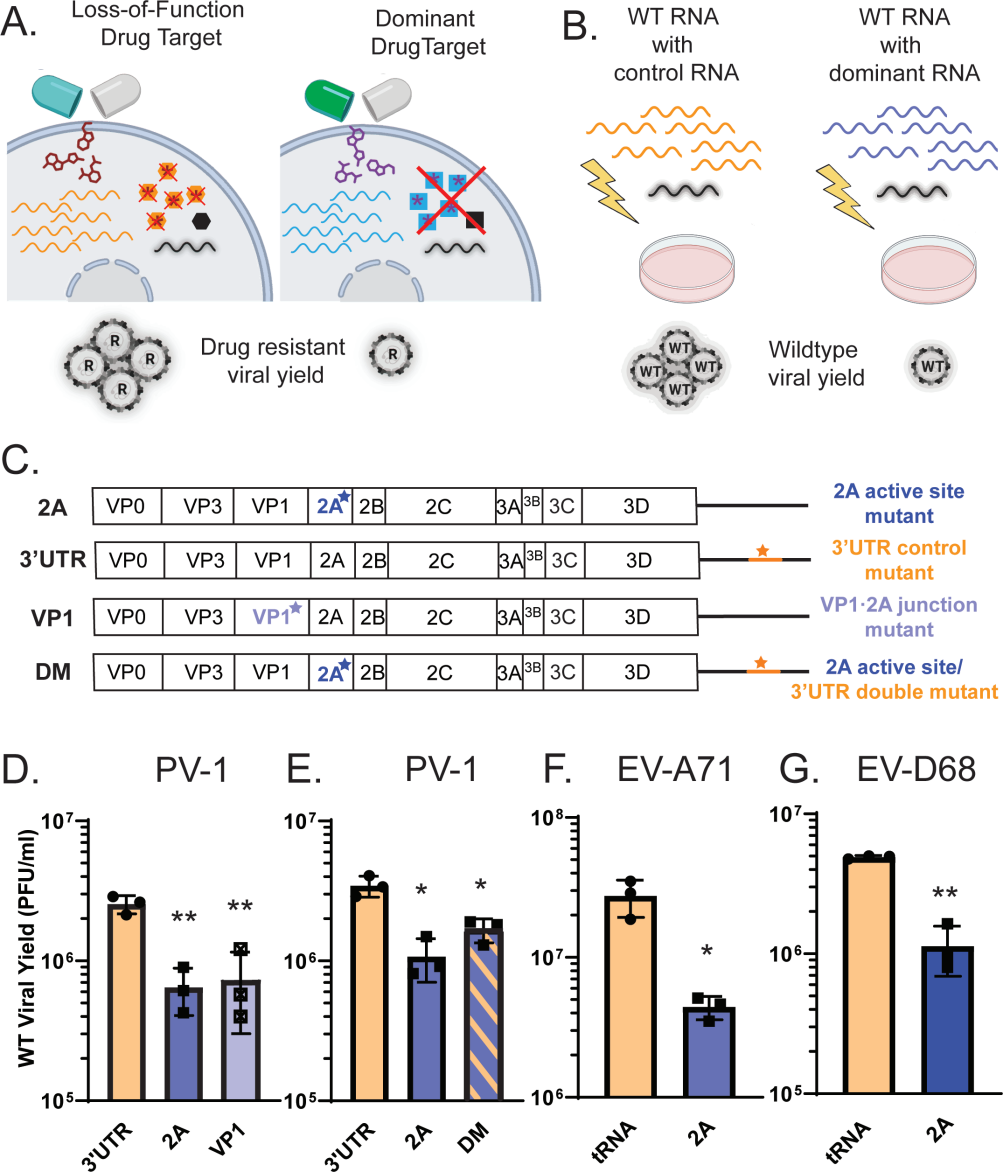
Inactive 2A is dominant over active 2A. (**A**) Model of dominant drug targets. In a loss-of-function drug target, viral products from drug-susceptible genomes (orange hexagons) have no effect on a newly arisen drug-resistant variant (black hexagon), so the drug-resistant variant replicates freely and takes over the population. For a dominant drug target, the drug-susceptible viral protein (blue square) has a toxic effect on drug-resistant viral products (black square), and the replication of the drug-resistant variant is suppressed. (**B**) Cotransfection experimental design to model a scenario in which a drug-resistant variant has arisen surrounded by drug-susceptible variants. WT viral genomes (black) are cotransfected with an excess of either control RNA (orange) or potentially dominant mutant RNA (blue) via electroporation. Yield of WT viral RNA is quantified. (**C**) Viral genomes used in cotransfection experiments. 3′ UTR is replication incompetent and known to be non-dominant and is therefore used as a control. (**D and E**) Cotransfection of WT poliovirus with indicated mutant RNA. 3′ UTR, 3′ UTR ΔGUA_3_ mutant. 2A, 2A C109R mutant. VP1, VP1 Y302P mutant. DM, 2A C109R 3′ UTR ΔGUA_3_ double mutant. (F) Contransfection of WT EV-A71 with 2A C110A mutant. tRNA used as a control for transfection of excess RNA. (**G**) Contransfection of WT EV-D68 with 2A C107R mutant. tRNA used as a control for transfection of excess RNA. All titers show the amount of WT virus formed in a mixed transfection with the indicated mutant. Poliovirus transfections were analyzed by one-way ANOVA, compared to the 3′ UTR condition. EV-A71 and EV-D68 transfections were analyzed by Student’s *t* test.

For most antivirals, the target simply loses function in the presence of the drug, and resistant variants will outcompete drug-susceptible variants. However, antivirals specifically blocking intra-molecular cleavages could suppress the emergence of drug-resistant variants if blocking such cleavages led to the accumulation of unprocessed precursors that interfered with the growth of more fit viruses (20, 49). We consider such cleavages to be potential dominant drug targets because inhibited drug-susceptible proteins can prevent the amplification of drug-resistant genomes, masking the resistance phenotype (Fig. 10A).

To test for dominance, we can recreate the circumstances of an intracellular quasispecies by cotransfecting cells with WT viral RNA and an excess of mutant viral RNA (Fig. 10B). In this case, the RNA with a mutation that inactivates the 2A protease is conceptually equivalent to an RNA genome whose 2A protein is rendered unfit by an antiviral. If infectious WT viral growth is inhibited by the products of these genomes, it suggests that 2A protease inhibition may lead to the generation of dominant inhibitors (Fig. 10B). Because RNA transfection efficiency is sensitive to the total amount of RNA, controls include the cotransfection of either a 10-fold excess of yeast tRNA or of a defective viral genome encoding a mutation in the 3′ untranslated region (UTR) known not to have a negative effect on WT growth.

In poliovirus, variants that fail to make the cleavage between VP1 and 2A, either because of a mutated protease active site or because of a mutated cleavage site, suppress the growth of WT viral genomes, while genomes with mutations in the 3′ UTR did not [Fig. 10C and D (50)]. However, 2A mutant genomes can undergo RNA replication both on their own (Fig. S3B) and in cotransfection with WT genomes (Fig. S3C), while 3′ UTR mutant genomes cannot. To test whether the genetic dominance of the 2A mutant genomes was due to their ability to amplify their RNA, a defective genome containing both 2A and 3′ UTR mutations was subjected to our cotransfection paradigm. The double mutant genomes were also found to be dominant inhibitors of WT viral growth (Fig. 10E), arguing that this mechanism of inhibition is distinct from that of defective interfering RNAs, which require genome replication to be inhibitory (51).

These experiments demonstrate that the *trans* dominance of poliovirus genomes encoding mutant 2A function correlates with a lack of cleavage at the VP1·2A junction. This correlation is extended by the observation that both EV-A71 and EV-D68 genomes with 2A active site mutations suppress the growth of the corresponding WT viruses (Fig. 10F and G). Thus, we hypothesize that the dominant inhibition of WT EV genomes by 2A mutant genomes is due to the accumulation of a VP1·2A containing precursor whose processing cannot be accomplished in *trans*.

## DISCUSSION

Although viral proteases perform a variety of tasks during infection, their most immediate task is to cleave themselves from the polyprotein in which they are embedded. The timing of viral polyprotein cleavages can play regulatory roles, controlling which viral protein products are present and at what proportions during infection. For many viruses, precursor proteins themselves play an essential role in the replication cycles (52), and some of them become inhibitory if not processed at the appropriate times (53–55).

Identifying the precise mechanisms of polyprotein cleavage for viral proteases with complex self-processing cascades is challenging. Expressing viral polyproteins in their entirety more closely mimics what happens during infection but is also more technically difficult, requiring extra steps such as immunoprecipitation to aid in data analysis (30, 56–58). Expressing truncated portions of the polyprotein, as has been done here, produces data that are simpler to interpret but that adhere less closely to the biological system. Utilizing either expression method, both dilution sensitivity and *trans* cleavage assays as described in this work have been used to investigate cleavage mechanisms in other viral systems such as alphaviruses (21, 22) and flaviviruses including dengue (20), West Nile virus (19), and hepatitis C virus (56–58).

In this study, we demonstrated that for poliovirus, EV-D68, and EV-A71, the polyprotein cleavage between VP1 and 2A occurs almost exclusively intra-molecularly. This is consistent with some (24, 25, 59) but not all (26, 27) previous work. We hypothesize that this rapid cleavage may represent a mechanism for the virus to quickly release the capsid proteins, which are large and difficult to fold (60), from the nascent polypeptide. Many picornavirus groups that do not encode a 2A protease still have a mechanism to rapidly separate VP1 and 2A, for example, by the addition of a ribosomal skipping mechanism in aphthoviruses (61, 62), potentially pointing to a strong selection pressure to ensure this separation.

Intra-molecular cleavages might be difficult to inhibit because of the constant proximity of the protease active site and its substrate. Consistent with the above hypothesis, we found that telaprevir inhibits the intra-molecular cleavage of the VP1·2A precursor with an IC_50_ that is more than 20-fold higher than that the IC_50_ for inter-molecular cleavage of host protein eIF4G (Fig. 7). This observation raises the question of whether the inhibition of EV-D68 viral growth by telaprevir is due to inhibition of the cleavage of the viral polyprotein or inhibition of host factors such as eIF4G.

The mechanism of 3C cleavage within the 3ABC polyprotein differs between poliovirus and EV-D68. We found that cleavage of the poliovirus 3ABC precursor occurs in *trans*. This is consistent with previous work in poliovirus, which assessed *trans* processing by adding infected cell lysates to uncleaved 3C precursors produced in translation extracts (32). The 3ABC precursor of EV-D68, on the other hand, is processed in *cis*. This has also been reported for the 3C protease encoded by EMCV as assessed via dilution sensitivity assay (34–36). Surprisingly, inhibition by rupintrivir of the intra-molecular cleavage of the EV-D68 3ABC precursor, with an IC_50_ of 140 nM, was more effective than inhibition of the inter-molecular cleavage of the poliovirus precursor, which displayed an IC_50_ of approximately 760 nM. Thus, it is feasible to effectively inhibit inter-molecular cleavages of viral polyproteins.

An important reason for targeting *cis* cleavages of viral polyproteins is illustrated by the cotransfection experiments in Fig. 10. Due to the error-prone nature of the viral RNA-dependent RNA polymerase, many variants arise spontaneously during EV infection and are subject to the crucible of selection. Although most will fail to propagate, some will encode useful features to the virus such as drug resistance. Our data argue that drugs blocking the intra-molecular cleavage between VP1 and 2A could potentially suppress the growth of resistant variants through the same mechanism that 2A mutant genomes suppress the growth of WT genomes in cotransfections. Indeed, it is possible that even partial inhibition of cleavage at the VP1·2A junction generates dominantly inhibitory precursors. Therefore, antivirals targeted toward intra-molecular polyprotein cleavages like those of EV 2A may both inhibit viral growth and suppress the selection of drug-resistant genomes.

## MATERIALS AND METHODS

### Chemicals and reagents

Telaprevir and rupintrivir were purchased from ChemSpace. zVAMfmk was custom-synthesized by Cortex Organics. Guanidine Hydrochloride was obtained from Sigma-Aldrich and suspended in water. All other compounds were suspended in DMSO. Rabbit monoclonal anti-eIF4G antibody was purchased from Cell Signaling Technologies.

### Protease constructs

Protease construct sequences from polio were derived from poliovirus Type 1 Mahoney, EV71 sequences from an EV-A71 4643, and EV-D68 US MO/14/18947. Relevant sequences were amplified via PCR with the addition of BamHI or EcoRI and XhoI cleavage sites. Protease constructs were cloned into pT7CFE1-Chis (Thermo Fisher) via T4 ligation (NEB). All mutations were made using QuikChange Lightning Mutagenesis Kit (Agilent). Complete list of constructs and primers can be found in Table S2. EV-D68 US/MO/14–18927 WT infectious clone and EV-A71 4643 WT, 2A C110A, and 3C C147A infectious clones were a gift of Jan Carette.

### Translation extracts

Translation reactions were performed using the TNT Quick Coupled Transcription/Translation Kit (Promega); 10- to 50-mL reactions were assembled according to kit directions and labeled using EasyTag L-[35S]-methionine (Perkin-Elmer). For pulse-chase reactions, after 20- to 30-minute L-methionine was added to a final concentration of 1 mM and the temperature was raised to 37°C to inhibit further translation. For dilution experiments, reactions were diluted with appropriate volumes of translation extract. At the indicated time point, aliquots were immediately mixed 1:1 with 2× Laemmli sample buffer. Samples were heated to 60°C for 5 minutes and separated by SDS/PAGE. Gels were prepared and imaged as in Constant et al. (20) and quantified using ImageStudio (LI-COR). For experiments evaluating compound activity, either DMSO or the compound of interest was added at the beginning of the reaction. DMSO comprised 1% of the final reaction volume unless otherwise noted. Inhibition was analyzed after 30 minutes in the chase phase.

### Western blots

For protease activity by *in vitro* translated protease, reactions were set up as above, with L-methionine substituted for EasyTag methionine. After 90 minutes, reactions were mixed 1:1 with 2× Laemmli sample buffer. To produce infected cell lysates, cells were infected at an multiplicity of infection of 10 with poliovirus Type 1 Mahoney, incubated for 5 hours, and lysed by the addition of 1× Laemmli buffer directly to cell monolayers. Both lysates and *in vitro* translation products were heated to 60°C for 5 minutes and separated by SDS/PAGE. Gels were transferred to polyvinylidene difluoride membrane using the Bio-Rad Trans-Blot Turbo system, and the membrane was dried overnight. After rehydration, membranes were blocked for 1 hour with Intercept Blocking Buffer TBS (LI-COR) and incubated overnight at 4°C with rabbit anti-eIF4G diluted 1:1,000 in Intercept Blocking Buffer. Membranes were then incubated for 1 hour with IRDye 800RD goat anti-rabbit antibody diluted 1:100,00 in Intercept Buffer and imaged on a LI-COR Odyssey Fc Imager.

### Viral RNA cotransfection

HeLa or rhabdomyosarcoma (RD) cells were electroporated as in Burrill et al. (63) with the following modifications. Cells were resuspended to a concentration of 8 × 10^6^ cells/mL in DPBS, and 1 mL was added to the electroporation cuvette. Fifty micrograms of total RNA was transfected into cells, at a ratio of 10:1 mutant or tRNA to WT viral RNA. Cells were electroporated with a Bio-Rad GenePulser Xcell using an exponential decay protocol (300 V, 500 µF, ∞Ω). Cells were recovered in DMEM and aliquoted as necessary. Poliovirus cotransfections were performed in HeLa cells, and EV-A71 and EV-D68 cotransfections were performed in RD cells.

### Plaque assay

After cotransfection, cells were aliquoted into four wells for technical replicates. After 12 (poliovirus), 16 (EV-A71), or 24 hours (EV-D68), cells were scraped into media and pelleted for 1 minute at 1,000 *g*. Cells were then resuspended in PBS++ and freeze-thawed three times. Viral titers were analyzed via plaque assay as described previously (64), using an Avicel overlay. Poliovirus and EV-A71 plaque assays were grown for 2 days at 37°C, and EV-D68 plaque assays were grown for 5 days at 34°C.

### Luciferase assay

After cotransfection, cells were aliquoted into eight wells. At the indicated time point, cells were scraped into media and pelleted for 1 minute at 1,000 *g*. After removal of supernatant, cells were resuspended in 100 µL of freshly prepared 1× Renilla Luciferase Assay Lysis Buffer (Promega). Samples were analyzed with the Renilla Luciferase Assay System (Promega) according to kit directions on a Promega GloMax Microplate reader.

### Statistical analysis

All experiments *n* = 3 unless otherwise noted. Quantifications were analyzed by two-way analysis of variance (ANOVA) with Dunnett’s multiple comparison tests unless otherwise noted. For *trans* cleavage assays, comparisons are to the WT self-processing. For dilution sensitivity assays, comparisons are to the undiluted reaction. *P < 0.05; **P < 0.01; ***P < 0.001; and ****P < 0.0001. IC_50_ calculations were performed by fitting the data to equation 1:


$$Y=100/\left(1+\frac{X}{{IC}_{50}}\right)$$

where *X* is the concentration of drug and *Y* is the percent cleavage. 100% cleavage is constrained to the amount of cleavage observed in the DMSO condition and 0% cleavage is defined as an uncleaved precursor.

## Data Availability

All plasmids are available on Addgene under the following IDs: for pT7CFE1_EVA71_VP12A_C110A, 212976; for pT7CFE1_EVA71_VP12A, 212977; for pT7CFE1_EVD68_3ABC_C147R, 212978; for pT7CFE1_EVD68_3ABC, 212979; for pT7CFE1_EVD68_3ABCD_C147R, 212980; for pT7CFE1_EVD68_3ABCD, 212981; for pT7CFE1_EVD68_VP12A_C107R, 212982; for pT7CFE1_EVD68_VP12A_N84T, 212983; for pT7CFE1_EVD68_VP12A, 212984; for pT7CFE1_polio_3ABC_C147R, 212985; for pT7CFE1_polio_3ABC, 212986; for pT7CFE1_polio_VP12A_C109R, 212987; and for pT7CFE1_polio_VP12A, 212988. Primary quantitative data are available on Zenodo.

## SUPPLEMENTAL MATERIAL

Figures S1-S3 and Tables S1 and S2 jvi.00928-23-s0001.docx Figure S1 (Gels with visible molecular weight markers), Figure S2 (2A-N84T mutant is not inhibited by telaprevir), Figure S3 (2A mutant poliovirus genomes can replicate), Table S1 (Expected molecular weights of cleavage products in translation reactions), and Table S2 (Primers used to generate expression and mutant constructs).

ASM does not own the copyrights to Supplemental Material that may be linked to, or accessed through, an article. The authors have granted ASM a non-exclusive, world-wide license to publish the Supplemental Material files. Please contact the corresponding author directly for reuse.
