# Supplementary material for: Differential inhibition of intra- and inter-molecular protease cleavages by antiviral compounds
Source: J Virol. 2023 Dec 4;97(12):e00928-23. doi: 10.1128/jvi.00928-23 (PMC10734437; doi:10.1128/jvi.00928-23)
Supplement: Figures S1-S3 and Tables S1 and S2 — Figure S1 (Gels with visible molecular weight markers), Figure S2 (2A-N84T mutant is not inhibited by telaprevir), Figure S3 (2A mutant poliovirus genomes can replicate), Table S1 (Expected molecular weights of cleavage products in translation reactions), and Table S2 (Primers used to generate expression and mutant constructs). [file jvi.00928-23-s0001.docx]

**SUPPLEMENTAL INFORMATION**

| **Protein** | **Molecular weight (kDa)** |
| --- | --- |
| VP12A | 50.05 |
| VP1 | 33.46 |
| 2A | 16.62 |
|  |  |
| 3ABCD | 84.7 |
| 3CD | 72.16 |
| 3D | 52.68 |
| 3ABC | 31.6 |
| 3BC | 21.61 |
| 3C | 19.48 |
| 3AB | 12.12 |

**Table S1.** Molecular weights of proteins identified in radioactive gels.

**
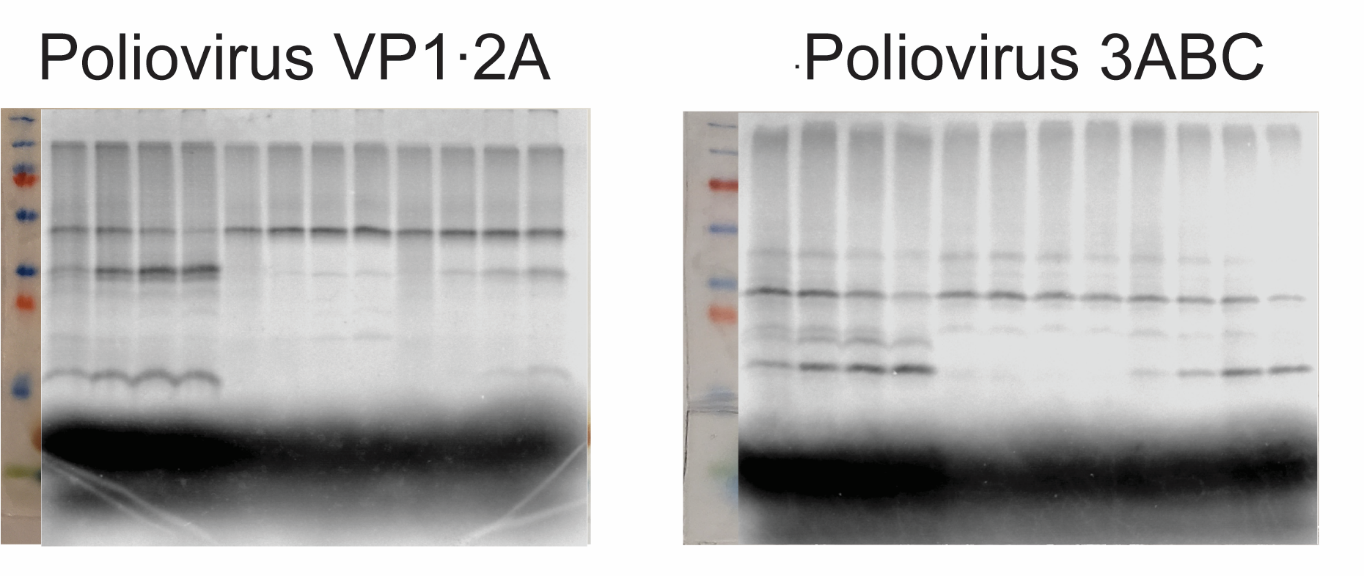
**

**Figure S1.** Visible molecular weight ladders were run with all gels and used to verify the sizes of the resultant bands. Representative examples of poliovirus VP1·2A (left) and 3ABC (right) are shown.


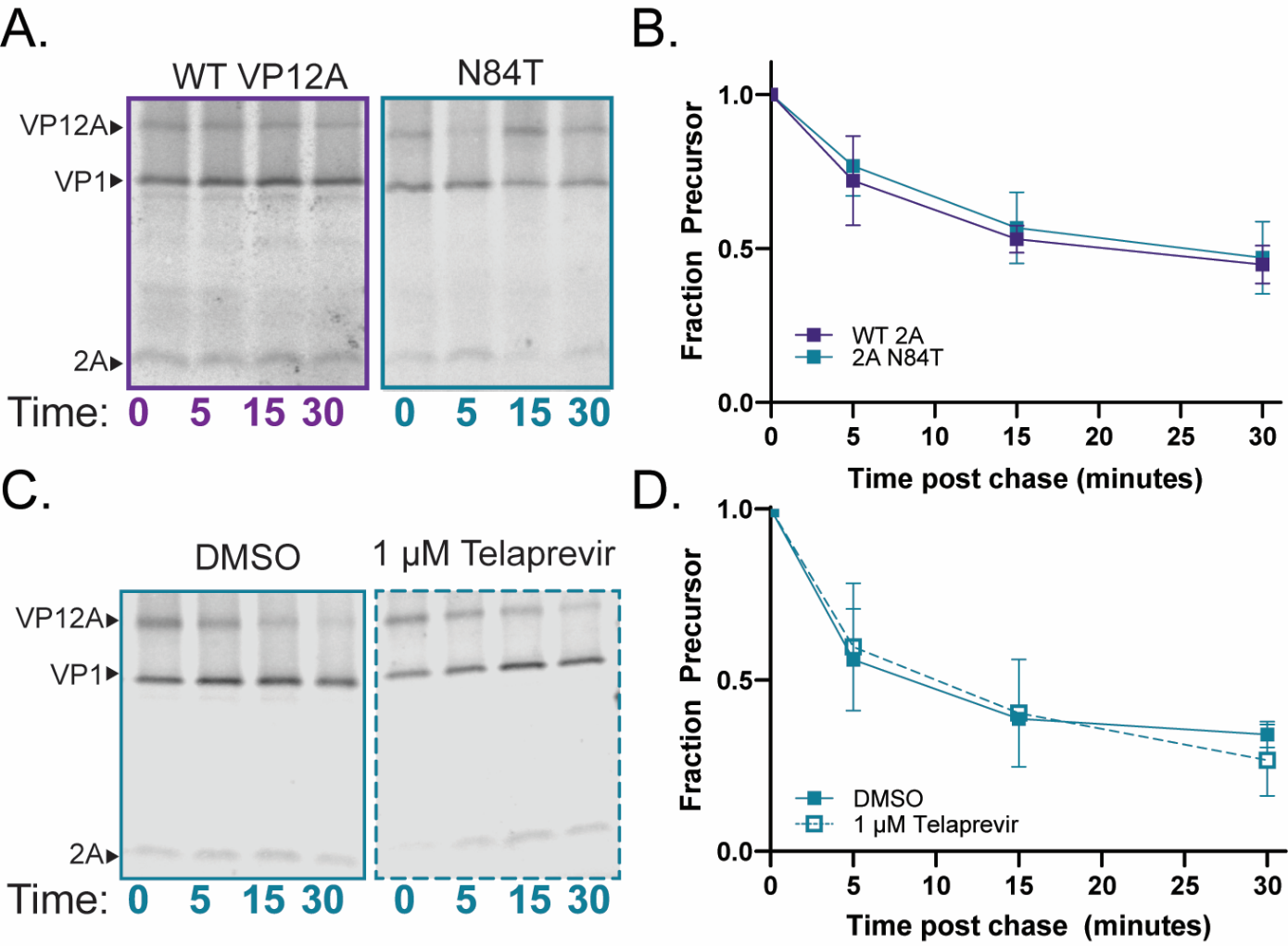


**Figure S2.** N84T mutant self-processing is not inhibited by telaprevir. (A and B) Representative gels (A) and quantification (B) of EV-D68 VP12A N84T mutant compared to wildtype processing. (C and D) Representative gels (C) and quantification (D) of N84T pulse chase in the presence of telaprevir. N84T mutation was previously characterized as conferring telaprevir resistance (1).


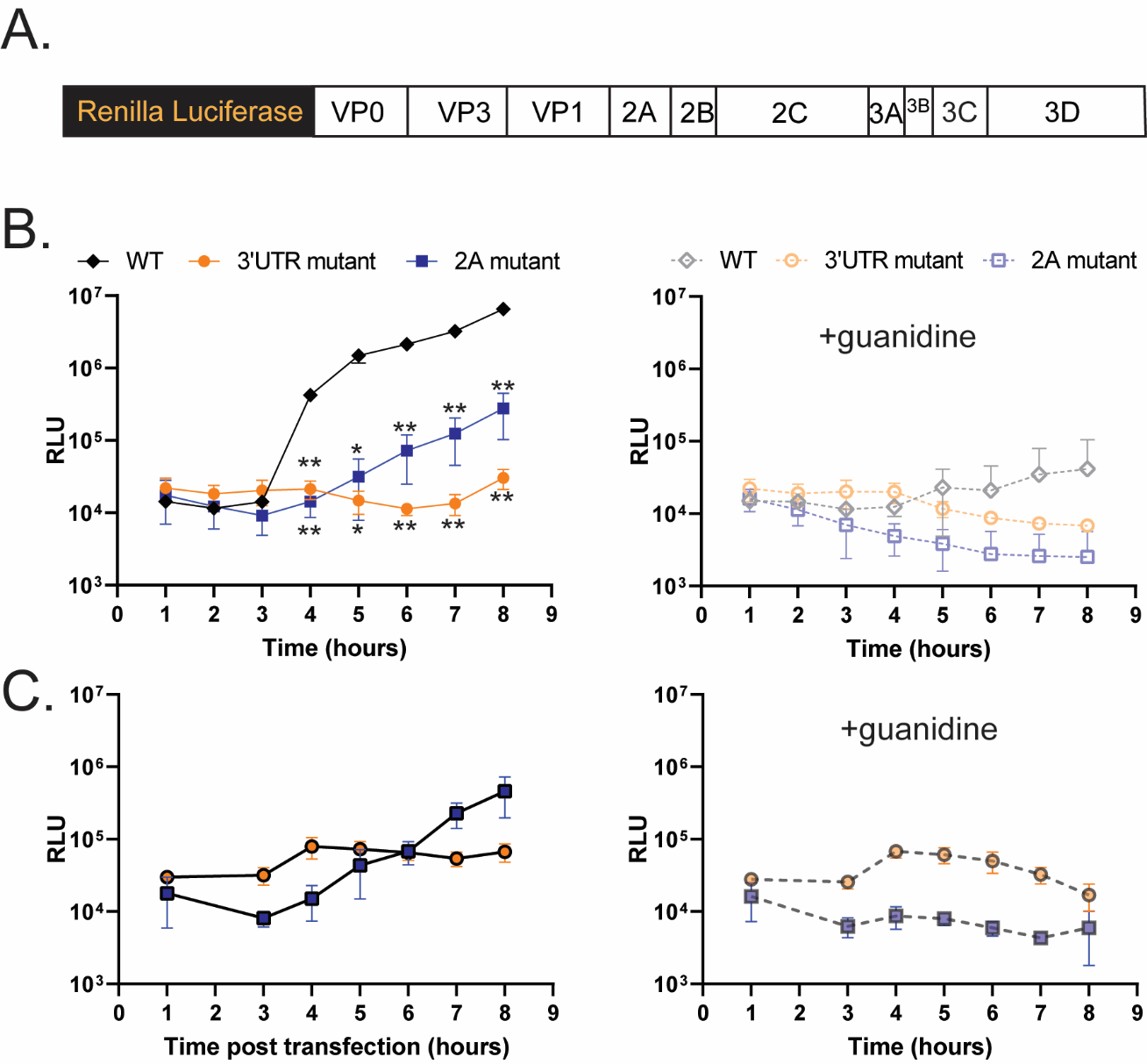


**Figure S3.** 2A mutant genomes can replicate. A) Renilla luciferase virus construct. Luciferase gene connected to the N terminus of P1 via a 3C cleavage site. B) Cells were singly transfected with a luciferase expressing viral RNA of the indicated genotype. Guanidine is an RNA replication inhibitor and allows us to assay translation separately from RNA replication. C) Cells were cotransfected with WT viral RNA and the indicated mutant luciferase construct.

| **Construct Name** | **Primer Sequence** |
| --- | --- |
| polio VP12A | atatggatccGGGTTAGGTCAGATGCTTGAAAGCATGATTG |
|  | atatctcgagTTGTTCCATGGCTTCTTCTTCGTAGGC |
| polio 3ABC | atatggatccGGACCACTCCAGTATAAAGACTTGAAAATTGACATCAAGAC |
|  | atatctcgagACTCTGAGTGAAGTATGATCGCTTCAGGG |
| EV-D68 VP12A | gactggatccCTAGACCATTTACATGCAG |
|  | atatctcgagTTGTTCCATAGCATCAGTATCTAACCATAGCAAATTCCTG |
| EV-D68 3ABC | atatgaattcGGGCCTCCACAGTTTAAAGAGATCAAAATTTCAGTC |
|  | atatctcgagTGTGTATCGGAAAAGTAAGAGTGTAGTAGCATTGC |
| EV-D68 3ABCD | atatgaattcGGGCCTCCACAGTTTAAAGAGATCAAAATTTCAGTC |
|  | atatctcgagGAACGAATCTAACCATTTCCGTCTAAGACTAGAGTATGC |
| EV-D68 2A only | atatgaattcGGTCCAGGCTTCGGAGGAGTTTTTG |
|  | atatctcgagTTGTTCCATAGCATCAGTATCTAACCATAGCAAATTCCTG |
| EV-D68 3C only | atatgaattcGGACCAGGGTTCGATTTTGCACAAG |
|  | atatctcgagTGTGTATCGGAAAAGTAAGAGTGTAGTAGCATTGC |
| EV-A71 VP12A | tactggatccGGAGATAGGGTGGCAGAT |
|  | tatactcgagCTGCTCCATGGCTTCATCATC |
|  |  |
| **Mutant Name** | **Primer Sequence** |
| polio 2A C109R | ccaggggatcgcggtggcatactc |
|  | gagtatgccaccgcgatcccctgg |
| polio 3C C147R | gatgactccaccacgctgtcctgctctgg |
|  | ccagagcaggacagcgtggtggagtcatc |
| Polio VP1 T301R | ggatctgaccaggtatggattcgg |
|  | ccgaatccatacctggtcagatcc |
| EV-D68 2A C107R | aaaccaccgcgatctcctgcttccgcagga |
|  | tcctgcggaagcaggagatcgcggtggttt |
| EV-D68 2A N84T | gctgggtaatattccgtttgttcaatccattgaatccctggg |
|  | cccagggattcaatggattgaacaaacggaatattacccagc |
| EV-D68 3C C147R | caagagctggccagcgtggtggtgtggtg |
|  | caccacaccaccacgctggccagctcttg |

**Table S2.** Primers used to make minimal protease constructs (top) and mutants (bottom). All primers ordered from IDT.

**SUPPLEMENTARY REFERENCES**

1. Musharrafieh R, Ma C, Zhang J, Hu Y, Diesing JM, Marty MT, Wang J. 2019. Validating Enterovirus D68-2Apro as an Antiviral Drug Target and the Discovery of Telaprevir as a Potent D68-2Apro Inhibitor. *J Virol* 93(7):e02221-18
